# Identification of Immune-Related Biomarkers for Sciatica in Peripheral Blood

**DOI:** 10.3389/fgene.2021.781945

**Published:** 2021-12-02

**Authors:** Xin Jin, Jun Wang, Lina Ge, Qing Hu

**Affiliations:** Department of Obstetrics and Gynecology, Shengjing Hospital of China Medical University, Shenyang, China

**Keywords:** sciatica, immunity, peripheral blood, biomarker, consensus clustering

## Abstract

**Objective:** Sciatica pertains to neuropathic pain that has been associated with inflammatory response. We aimed to identify significant immune-related biomarkers for sciatica in peripheral blood.

**Methods:** We utilized the GSE150408 expression profiling data from the Gene Expression Omnibus (GEO) database as the training dataset and extracted immune-related genes for further analysis. Differentially expressed immune-related genes (DEIRGs) between healthy controls and patients with sciatica were selected using the “limma” package and verified in clinical specimens by quantitative reverse transcription PCR (RT-qPCR). A diagnostic immune-related gene signature was established using the training model and random forest (RF), generalized linear model (GLM), and support vector machine (SVM) models. Sciatica patient subtypes were identified using the consensus clustering method.

**Results:** Thirteen significant DEIRGs were acquired, of which five (*CRP, EREG, FAM19A4, RLN1*, and *WFIKKN1*) were selected to establish a diagnostic immune-related gene signature according to the most appropriate training model, namely, the RF model. A clinical application nomogram model was established based on the expression level of the five DEIRGs. The sciatica patients were divided into two subtypes (C1 and C2) according to the consensus clustering method.

**Conclusions:** Our research established a diagnostic five immune-related gene signature to discriminate sciatica and identified two sciatica subtypes, which may be beneficial to the clinical diagnosis and treatment of sciatica.

## Introduction

Sciatica is a common clinical syndrome caused by irritation of the sciatic nerve and consists of two types: spinal and extra-spinal (Ailianou et al., 2012). Sciatica is diagnosed mainly based on history and physical examination. A history of leg pain more intense than back pain or pain below the knee should raise suspicion of sciatica. Inquiries relating to onset and distribution of pain and associated symptoms such as tingling sensation, numbness, or muscle weakness in the legs are also helpful (Jensen et al., 2019). There is no specific test for sciatica (Stynes et al., 2018). A recent cohort study proposed clinical criteria for unilateral leg pain, monoradicular distribution of pain, positive straight leg raise test at <60° (or femoral stretch test), unilateral motor weakness, and asymmetric ankle reflex to predict sciatica caused by lumbar disc herniation. The patients should be excluded serious pathology such as cancer, trauma, and infection (Genevay et al., 2017). The sciatic nerve is generally visualized using magnetic resonance imaging (MRI) (Ailianou et al., 2012). Spinal sciatica is mainly caused by single-level lumbar disc herniation at the L4/5 or L5/S1 level (Wang et al., 2021a). Current treatment methods for sciatica mainly include conservative treatment, exercise, manual therapy, and surgery (Jensen et al., 2019). Conservative treatment encourages patients to remain active and avoid bed rest (Stochkendahl et al., 2018). Exercise reduces the intensity of leg pain in the short term, and manual therapy such as spinal mobilization can be offered alongside exercise and may be provided by manual therapists, physiotherapists, and chiropractors based on local practice (Stochkendahl et al., 2018). Imaging should confirm lumbar disc herniation at the nerve root level corresponding with findings on clinical examination. Open microdiscectomy for removal of disc herniation is the most common procedure and is minimally invasive (Jensen et al., 2019). However, regardless of the treatment method, the resulting clinical effect is not stable (Jensen et al., 2019). Therefore, it is of clinical significance to screen effective diagnostic and treatment methods for sciatica.

Sciatica is caused by compression of the nerve root after lumbar disc herniation. Except for a few acute injuries, most of these are caused by chronic strain of soft tissue, which leads to degeneration of ligament, facet joint process, and intervertebral ring fiber, making it lose its normal protection. Sciatica is not only caused by mechanical compression or drawing but also by chemical stimulation and immune response (Sun et al., 2013; Wang et al., 2021b). Stimulation of the chemicals in the nucleus pulposus, including glycoproteins, β-proteins, and histamines, can lead to acute chemical neuritis, where inflammatory substances come into contact with the body’s immune system such as antigens, triggering an autoimmune response that leads to disc lesions and sciatica. Sciatic menstrual pain can mostly be relieved with acupuncture treatment. This relief does not depend on whether the protruded nucleus pulposus can be recovered but on the relief of adhesion, edema, and aseptic inflammation around the nerve root and the recovery of the normal immune response of the body (Li et al., 2018).

Individuals with sciatica present inflammatory and immune characteristics that may be detected in peripheral blood (Wang et al., 2021a). In our study, we screened for differentially expressed immune-related genes (DEIRGs) between healthy controls and sciatica patients using the “limma” package and verified our results in clinical specimens by RT-qPCR analysis. A random forest (RF) analysis was conducted to predict the potential diagnostic value of the DEIRGs in sciatica. Finally, consensus clustering was performed, which classified sciatica patients into two subtypes, which may be beneficial to individualized treatment of sciatica.

## Materials and Methods

### Data Source

The training cohort GSE150408 containing 17 healthy controls and 42 sciatica patients was acquired from the Gene Expression Omnibus (GEO) database (http://www.ncbi.nlm.nih.gov/geo) (Wang et al., 2021a). Immune-related genes (IRGs) were downloaded from the ImmPort database (https://immport.niaid.nih.gov) (Huang et al., 2020). We extracted 2,499 IRGs from the gene expression profile data of the GSE150408 dataset for further analysis.

### Specimen Collection

From January 2019 to December 2020, we recruited eight sciatica patients (age range: 30–52 years old; average age: 40 years old) and eight healthy volunteers (age range: 18–28; average age: 23). The inclusion criteria for sciatica patients were as follows: (1) The patient’s symptoms and examination suggest sciatica; and (2) single-level lumbar disc herniation at the L4/5 or L5/S1 level resulting in corresponding nerve root compression, as detected by MRI. The patients were spinal sciatica but not extra-spinal sciatica. The exclusion criteria for sciatica patients were as follows: (1) Sciatica concomitant to neuropathy, other spinal diseases, cardiovascular disease, infections, metabolic disease, rheumatism, dementia or mental health disorders, or a history of surgery, or neoplasms; and (2) patients who have received any medication in the past 3 months. Between 7:00 and 7:30 a.m., 10 ml of fasting peripheral blood was collected from the left median cubital vein of each participant. This study was approved by the ethics committee of ShengJing Hospital of China Medical University (Shenyang, Liaoning province, China), and informed consent was obtained from all patients and healthy participants. In addition, all methods were performed in accordance with relevant guidelines and regulations.

### Identification of DEIRGs

The “limma” package was used to screen DEIRGs between 17 healthy controls and 42 sciatica patients with the inclusion criteria log∣FC∣ > 0.2 and *p* < 0.001. The “ggplot2” package was used to draw a histogram, and the “ComplexHeatmap” package was employed to draw a heat map depicting the expression of DEIRGs between healthy controls and sciatica patients (Wen et al., 2020; Ritchie et al., 2015).

### RT-qPCR Analysis

RT-qPCR analysis was performed to verify the expression level of DEIRGs between eight healthy controls and eight sciatica patients from ShengJing Hospital of China Medical University. Total RNA was extracted from blood and purified according to the manufacturer’s protocol of the PX Blood RNA Kit (Omega Bio-Tek Inc.). RNA samples were reverse-transcribed into cDNA using the ReverTra Ace qPCR kit (Toyobo), and cDNA was amplified according to RT-qPCR methods. The mRNA expression levels of DEIRGs were calculated by the 2^−ΔΔCT^ method, and GAPDH was used as internal reference (Wang et al., 2019). The sequences of the primers used in RT-qPCR analysis are shown in Supplementary Table S1.

### Construction of Random Forest, Generalized Linear Model, and Support Vector Machine Models

The “pacman” package in R software was used to establish RF, GLM, and SVM models using the DEIRGs as explanatory variables and sciatica patients as response variables. A receiver operating characteristic (ROC) curve, reverse cumulative distribution of ∣residual∣, and boxplots of ∣residual∣ were plotted to select the most appropriate model as the training model. Finally, the “mlbench” and “caret” packages were used to rank the explanatory variables by importance. A cross curve was drawn to select explanatory variables (Wang et al., 2020; Pan et al., 2021).

### Construction of the Nomogram Model

A nomogram model was established based on the selected explanatory variables using the “rms” package. According to the characteristics of each variable of the patient, the score of each item was obtained by projecting upward to the small scale (points). The score of each item was added up to obtain the total value. The higher the total value, the greater the probability of sciatica. To verify the performance of the model, the calibration curve, decision curve analysis (DCA) curve, and clinical impact curve were drawn (Yu and Zhang, 2019).

### Consensus Clustering of DEIRGs

To investigate the role of DEIRGs in sciatica, we used “ConsensusClusterPlus” in R software to divide the sciatica patients into different subgroups according to the expression of DEIRGs. The grouping principles were as follows: (1) the cumulative distribution function (CDF) is small and increased slowly; and (2) there is no small cluster or cross cluster in the cluster data (Wilkerson and Hayes, 2010; Zhang et al., 2020).

### Statistical Analysis

All of the statistical analyses were performed using R software (version 3.6.2). The expression of DEIRGs was compared between sciatica patients and healthy controls as well as different sciatica subgroups using the Wilcoxon’s test. The correlation between DEIRGs was determined by Spearman’s correlation analysis.

## Results

### Landscape of 13 DEIRGs in Sciatica Patients

**FIGURE 1:**
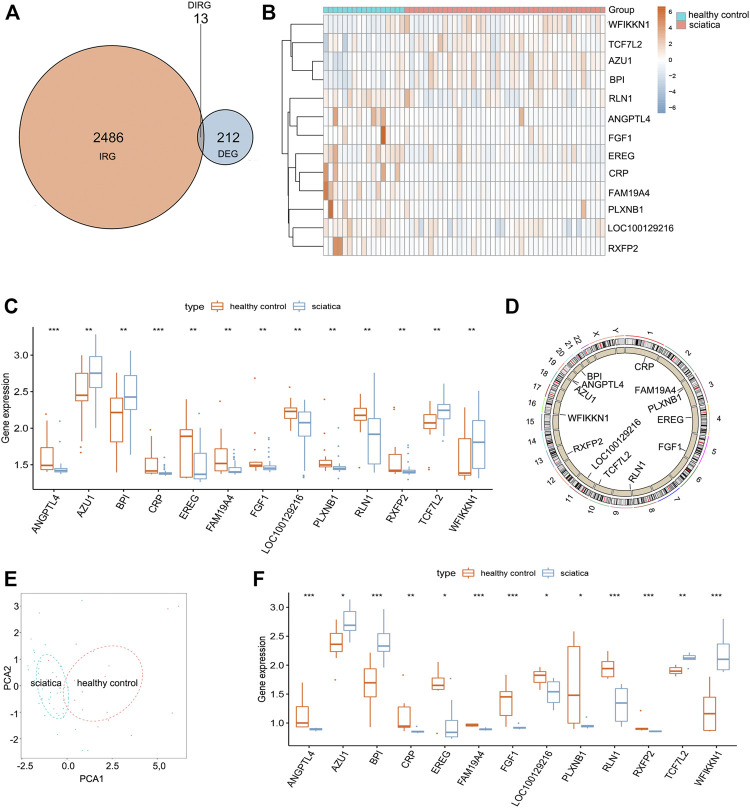
Landscape of 13 differentially expressed immune-related genes (DEIRGs) in sciatica. **(A)** Identification of 13 DEIRGs. **(B)** Heat map showing the expression of 13 DEIRGs between sciatica patients and healthy controls in the GSE150408 dataset. **(C)** Histogram showing the expression of 13 DEIRGs between sciatica patients and healthy controls in the GSE150408 dataset. **(D)** Loop graph showing the chromosomal positions of the 13 DEIRGs. **(E)** PCA based on the expression of the 13 DEIRGs can distinguish sciatica patients from healthy controls. **(F)** Histogram showing the expression of 13 DEIRGs between eight sciatica patients and eight healthy controls based on the results of RT-qPCR analysis.

**FIGURE 2:**
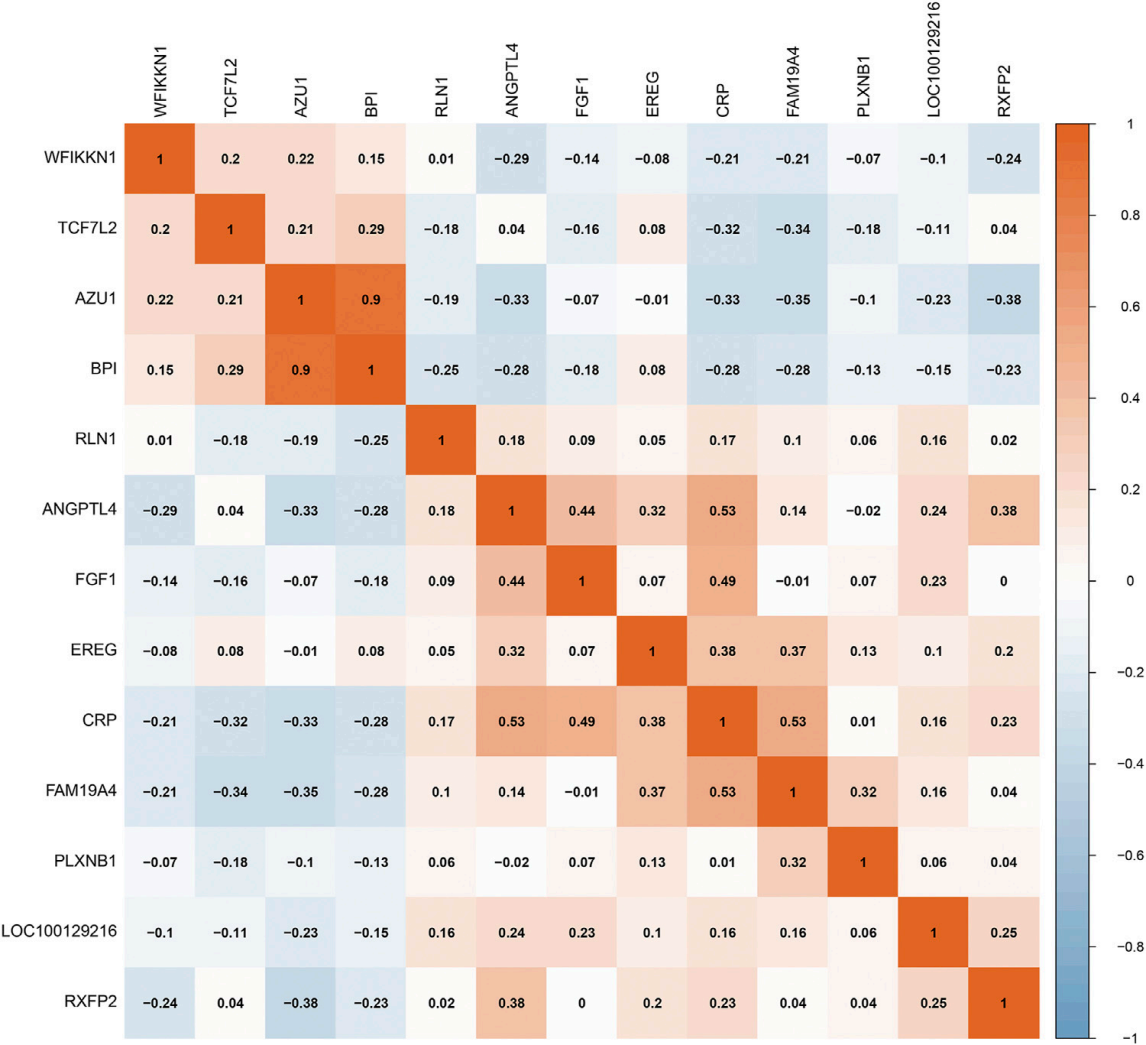
Spearman’s correlation analysis of 13 DEIRGs.

A total of 212 differentially expressed genes (DEGs) between sciatica patients and healthy controls were obtained based on the inclusion criteria log∣FC∣ > 0.2 and *p* < 0.001. Crossing the 212 DEGs with 2,499 IRGs, 13 DEIRGs were acquired (Figure 1A). Figures 1B,C depict the expression levels of the 13 DEIRGs between sciatica patients and healthy controls. AZU1, BPI, TCF7L2, and WFIKKN1 were upregulated in sciatica patients compared with healthy controls, whereas ANGPTL4, CRP, EREG, FAM19A4, FGF1, LOC100129216, PLXNB1, RLN1, and RXFP2 were downregulated. Figure 1D shows the chromosomal position of the 13 DEIRGs. Principal component analysis (PCA) based on the expression of the 13 DEIRGs distinguished sciatica patients from healthy controls (Figure 1E). Finally, we successfully verified the expression of the 13 DEIRGs between eight healthy controls and eight sciatica patients (Figure 1F). Spearman’s correlation analysis of the 13 DEIRGs revealed that AZU1 and BPI had the highest positive correlation, while AZU1 and RXFP2 exhibited the highest negative correlation (Figure 2).

### Construction of the RF Model

**FIGURE 3:**
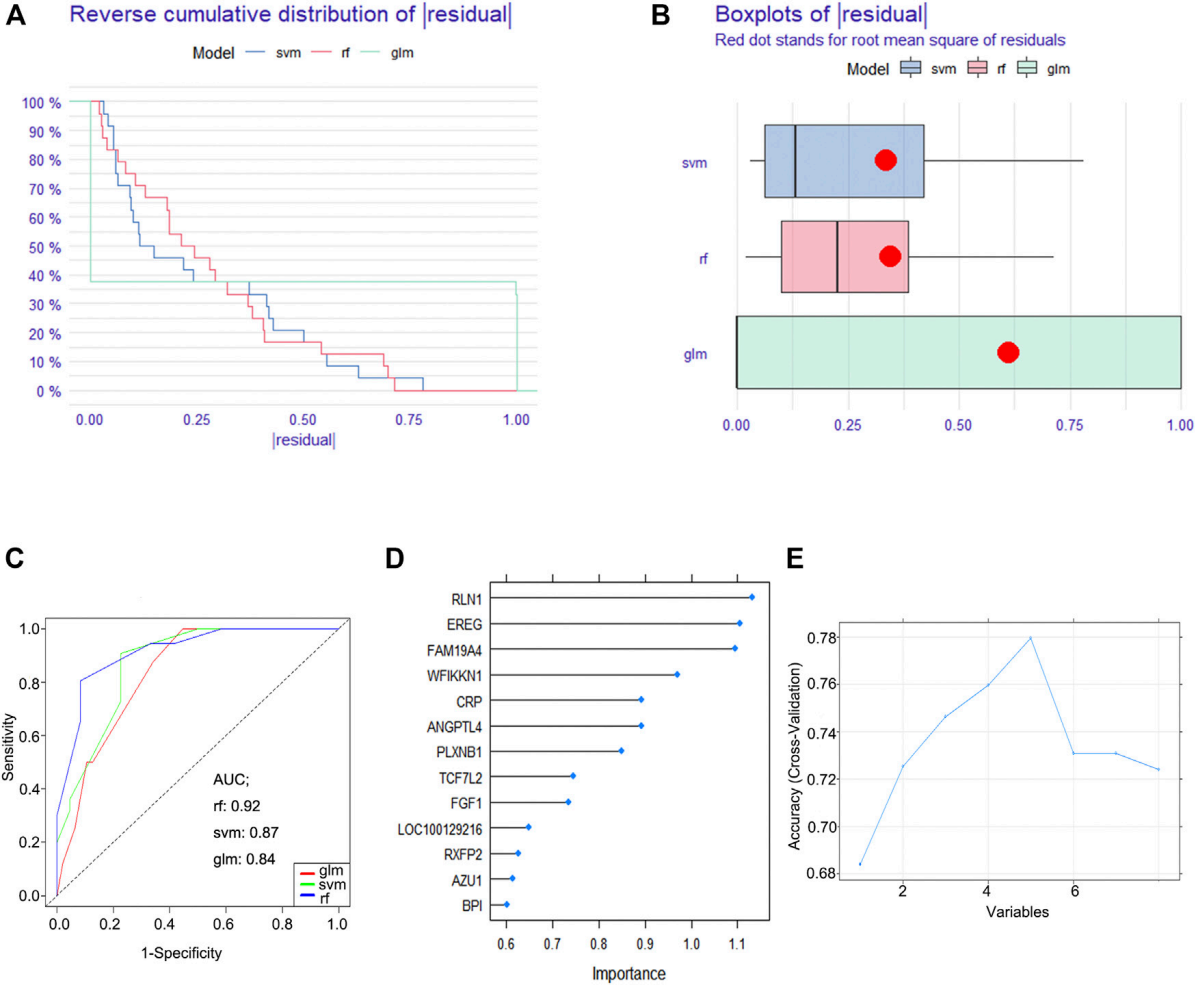
Construction of a diagnostic immune-related gene signature. **(A)** Cumulative distribution of ∣residual∣ in RF, GLM, and SVM models. **(B)** Boxplots of ∣residual∣ in RF, GLM, and SVM models. **(C)** ROC curve and AUC value of RF, GLM, and SVM models. **(D)** The importance of explanatory variables ranked by the RF model. **(E)** Cross curve showing the accuracy of the RF model.

To establish a diagnostic immune-related gene signature, RF, GLM, and SVM models were separately established. Reverse cumulative distribution of ∣residual∣ and boxplots of ∣residual∣ revealed that the RF model has the lowest residual distribution compared with the GLM and SVM models (Figures 3A,B). ROC curve analysis indicated that the AUC value of the RF model was higher than that of the GLM and SVM models (Figure 3C). Based on these results, we deemed the RF model to be the most appropriate training model. We then ranked the explanatory variables by importance (Figure 3D). Cross curve analysis indicated that the accuracy of the training model was superior when the first five explanatory variables (RLN1, EREG, FAM19A4, WFIKKN1, and CRP) were used to establish a diagnostic immune-related gene signature (Figure 3E).

### Construction of the Nomogram Model

**FIGURE 4:**
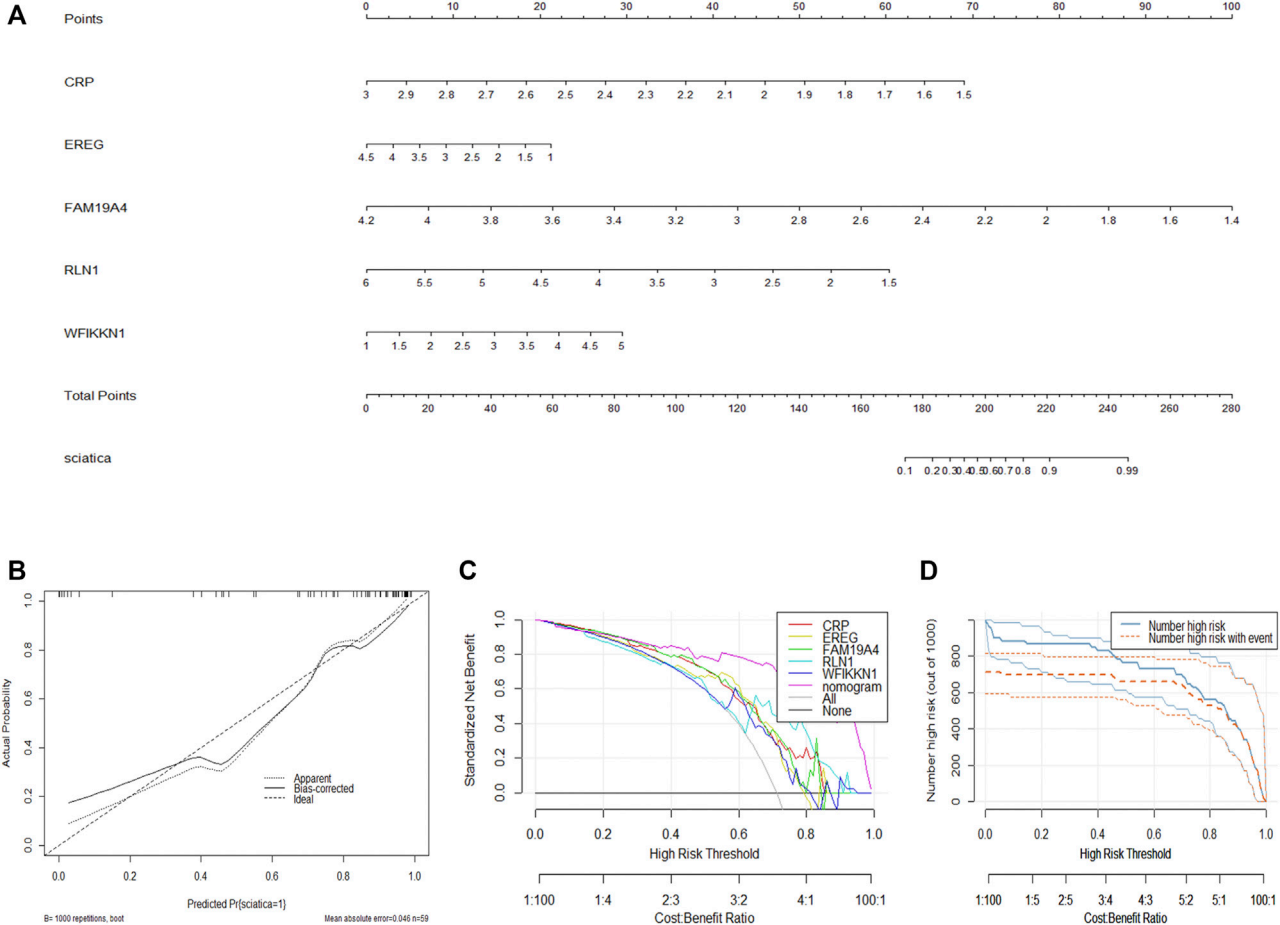
Construction of the nomogram model. **(A)** Construction of the nomogram model based on the selected DEIRGs (RLN1, EREG, FAM19A4, WFIKKN1, and CRP). **(B)** Calibration curve showing the diagnostic power of the nomogram model. **(C)** DCA showing that the nomogram model has higher clinical application value than individual DEIRGs. **(D)** Clinical impact curve showing that the diagnostic power of the nomogram model is relatively high.

To facilitate the diagnosis of sciatica by clinicians using the selected DEIRGs (RLN1, EREG, FAM19A4, WFIKKN1, and CRP), we constructed a nomogram model (Figure 4A). Calibration curves showed that the positive rate of sciatica diagnosed by the nomogram model was consistent with the actual positive rate (Figure 4B). DCA showed that although both the nomogram model and individual DEIRG produced net benefits, the net benefits generated by the nomogram model were significantly higher than those generated by individual DEIRGs, suggesting that the nomogram model is of higher clinical application value than individual DEIRGs (Figure 4C). Clinical impact curve analysis suggested that the diagnostic power of the nomogram model is relatively high (Figure 4D).

### Identification of Sciatica Subgroups by Consensus Clustering

**FIGURE 5:**
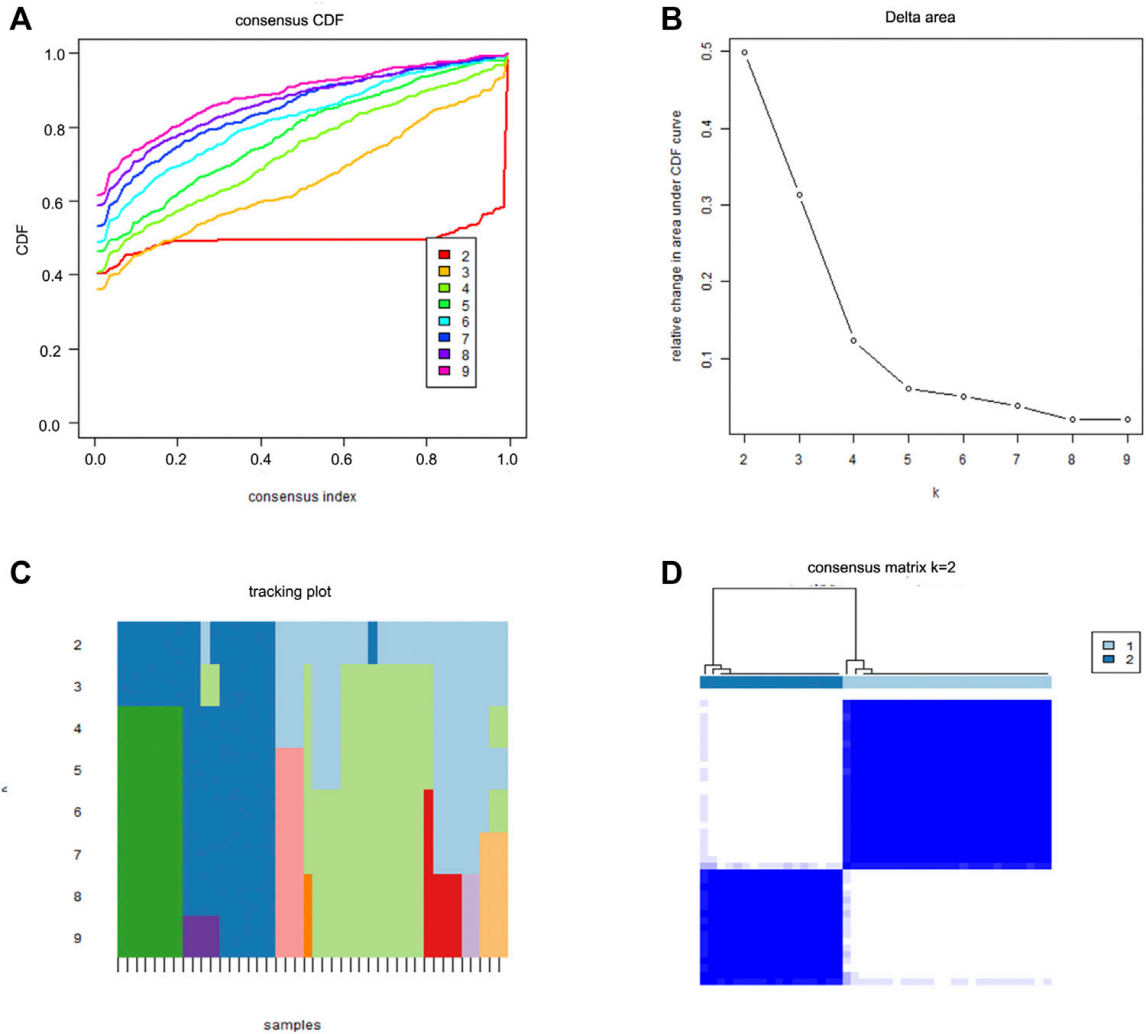
Two sciatica subgroups obtained by consensus clustering. **(A)** CDF curve for *k* = 2–9. **(B)** The delta area score of the CDF curve for *k* = 2–9. **(C)** Tracking plot for *k* = 2–9. **(D)** The matrix heat map was separated when *k* = 2.

**FIGURE 6:**
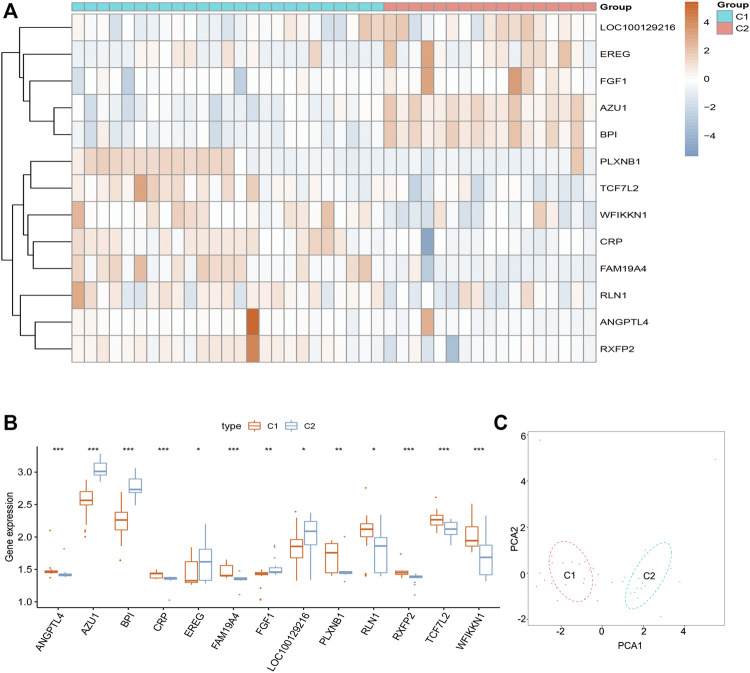
Landscape of two sciatica subgroups in the GSE150408 dataset. **(A)** Heat map showing the expression of 13 DEIRGs between the C1 and C2 groups in the GSE150408 dataset. **(B)** Histogram showing the expression of 13 DEIRGs between the C1 and C2 groups in the GSE150408 dataset. **(C)** PCA based on the expression of the 13 DEIRGs can distinguish between the C1 and C2 groups.

**FIGURE 7:**
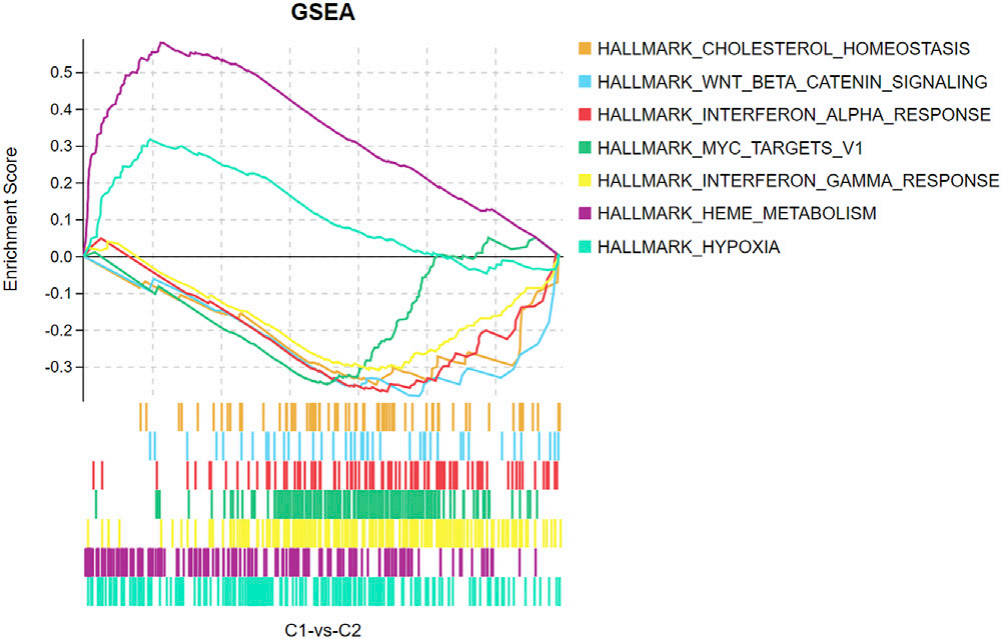
GSEA enrichment analysis showing functional enrichment in the C1 and C2 groups.

We divided the sciatica patients into several subgroups using the consensus clustering method to explore the role of the 13 DEIRGs in sciatica. The results show that when *k* = 2, 3, and 4, the CDF value gradually increases, and when *k* = 4, the CDF value is small (Figures 5A,B). However, the correlations between groups were high when the sciatica patients were divided into three or four groups. Therefore, we divided the sciatica patients into two subgroups (C1 and C2, Figures 5C,D). Figures 6A,B shows that AZU1, BPI, EREG, and LOC100129216 were upregulated in the C2 group compared with the C1 group, whereas TCF7L2, WFIKKN1, ANGPTL4, CRP, FAM19A4, FGF1, PLXNB1, RLN1, and RXFP2 were downregulated. To determine whether our classification is correct, PCA was performed based on the expression profiles of the 13 DEIRGs. The results showed that there are obvious differences between the C1 and C2 groups (Figure 6C). Gene Set Enrichment Analysis (GSEA) revealed that the patients in the C1 group were mainly enriched in heme-metabolism and hypoxia, while those in the C2 group were mainly enriched in cholesterol homeostasis, WNT-beta-catenin signaling, interferon alpha response, MYC targets, and interferon gamma response (Figure 7).

## Discussion

Sciatica is a common neuropathic pain that currently lacks effective diagnostic biomarkers and treatment methods. Inflammatory and immune responses play an important role in pathophysiological processes associated with neuropathic pain following peripheral nerve injury (Kim and Moalem-Taylor, 2011). In our study, we comprehensively explored the role of immune-related genes in sciatica, providing evidence for effective diagnosis and individual treatment of sciatica.

Thirteen DEIRGs (AZU1, BPI, TCF7L2, WFIKKN1, ANGPTL4, CRP, EREG, FAM19A4, FGF1, LOC100129216, PLXNB1, RLN1, and RXFP2) between sciatica patients and healthy controls were obtained using the inclusion criteria log∣FC∣ > 0.2 and *p* < 0.001. AZU1, BPI, TCF7L2, and WFIKKN1 were upregulated in sciatica patients, while ANGPTL4, CRP, EREG, FAM19A4, FGF1, LOC100129216, PLXNB1, RLN1, and RXFP2 were downregulated. AZU1, BPI, CRP, FAM19A4, LOC100129216, TCF7L2, and WFIKKN1 belong to the category of antimicrobials. ANGPTL4, PLXNB1, and RXFP2 are cytokine receptors. AZU1, EREG, FGF1, and RLN1 pertain to cytokines, while PLXNB1 belongs to the family of chemokine receptors. To our knowledge, AZU1, BPI, TCF7L2, WFIKKN1, ANGPTL4, EREG, FAM19A4, FGF1, LOC100129216, PLXNB1, RLN1, and RXFP2 have not been implicated in sciatica. C-reactive protein (CRP), as an indicator of inflammation, has been reported to be elevated in the serum of sciatica patients (Uher and Bob, 2013). Then, RF, GLM, and SVM models were separately established to establish a diagnostic immune-related gene signature based on 13 DEIRGs. RF is an integrated learning algorithm that combines different decision trees. Among the decision trees that constitute an RF model, each tree is an independent set generated based on random samples. Each tree independently learns and predicts, and the final result is determined by the mean value of all decision trees (Pavlov, 1997; Jeong et al., 2020). The GLM model is an extension of the linear model that establishes the relationship between the mathematical expected value of the response variables and the predictive variables of the linear combination through the coupling function (Blough et al., 1999; Qu and Luo, 2015). SVM is a discriminant classifier defined by the classification hyperplane. The model is trained with labeled training samples, and then, the test samples are classified by the output of the optimal hyperplane (Huang et al., 2018). The RF model has the lowest residual distribution and the highest AUC value compared with the GLM and SVM models, indicating that the RF model is the most appropriate training model. Finally, a diagnostic immune-related gene signature and a nomogram model, which includes five explanatory variables (RLN1, EREG, FAM19A4, WFIKKN1, and CRP), were established. The DCA curve and clinical impact curve analyses suggested that the nomogram model has significant clinical diagnostic benefits.

To investigate the role of 13 DEIRGs in sciatica, “ConsensusClusterPlus” was used to divide the sciatica patients into two subgroups according to the expression of the 13 DEIRGs. AZU1, BPI, EREG, and LOC100129216 were upregulated in the C2 group, while TCF7L2, WFIKKN1, ANGPTL4, CRP, FAM19A4, FGF1, PLXNB1, RLN1, and RXFP2 were downregulated. GSEA revealed that the patients in the C1 group were mainly enriched with DEGs related to heme-metabolism and hypoxia, while the C2 group was mainly enriched with DEGs associated with cholesterol homeostasis, WNT-beta-catenin signaling, interferon alpha response, MYC targets, and interferon gamma response. PCA analysis based on the expression profiles of the 13 DEIRGs can effectively distinguish the C1 group from the C2 group. Our results indicate that sciatica patients have two completely different subtypes. In future studies, we will collect more clinical characteristics to distinguish between the two completely different sciatica subtypes through differential analysis, which may provide a basis for the clinical diagnosis and precise treatment of sciatica patients. In addition, it is also interesting to explore the 13 DEIRGs in relation to inflammatory blood markers, combined with MRI findings, for the diagnosis of sciatica.

## Conclusion

We have constructed a diagnostic immune-related gene signature based on five explanatory variables and identified two different sciatica subtypes that may be utilized in the diagnosis and individualized treatment of sciatica.

## Data Availability

The original contributions presented in the study are included in the article/Supplementary Material. Further inquiries can be directed to the corresponding author.
