# Supplementary material for: Identification of Immune-Related Biomarkers for Sciatica in Peripheral Blood
Source: Front Genet. 2021 Dec 2;12:781945. doi: 10.3389/fgene.2021.781945 (PMC8677837; doi:10.3389/fgene.2021.781945)
Supplement: Supplementary file 1 [file Table1.DOCX]

| Gene | Sequences of the primers |
| --- | --- |
| \| ANGPTL4 \| \| --- \| | F:5' TCTCTGGAGGCTGGTGGTTTGG 3'  R:5’ CGATCTCAGGAGGCAACGCATG 3’ |
| CRP | F:5' CGCCTGAGAATGGAGGTAAAGTGTC 3’  R:5’ GAGACTGAAGAGCCCTGTGAATGTG 3’ |
| EREG | F:5' AGGAGGATGGAGATGCTCTGTGC 3’  R:5’ ATGTGGAACCGACGACTGTGATAAG 3’ |
| FAM19A4 | F:5' AAGTGCTGCCAGATTACTCAGGTTG 3’  R:5’ CAGAGAGGATGTCAAATGGGTGGTG 3’ |
| FGF1 | F:5’ CCTTCTGATGGTGAATGGGAACTCC 3’  R:5' GAATGTGCTGGTCGCTCCTGTC 3’ |
| LOC100129216 | F:5’ CTCGTAGGCAACACCACCATCATC 3’  R:5' CCCAGACCCAGGAACAGGAAGAG 3’ |
| PLXNB1 | F:5' GATGGGACCTTTGAGCACCTGTATG 3'  R:5’ CCTCGTCTCCTCTCGGCTGATG 3’ |
| RLN1 | F:5’ GCCTGTTCTTGTTCCACCTGCTAG 3’  R:5’ GCTCTGGTAATGATGGTTGCCTCTC 3’ |
| TCF7L2 | F:5’ CCCACCACATCATACGCTACACAC 3’  R:5’ CGCTTGCTCTTCTCTGGACAGTG 3’ |
| WFIKKN1 | F:5’ CGAGTGTAGCAGGGACCAGGAC 3’  R:5’ TAGCAGCGGTTGTAGTAGGTGAGG 3’ |
| AZU1 | F:5’ ATCAGCAGCATGAGCGAGAATGG 3’  R:5’ CAAACCTGGGAAAACGGGAGAGAC 3’ |
| BPI | F:5’ ATCAAGATCAGCGGGAAATGGAAGG 3’  R:5’ GCTCGGAGGATACAGAATTGGTCAC 3’ |
| RXFP2 | F:5' GACGGACGGCATTTCTTCATTTGAG 3'  R:5’ CTCTCCATCCACAGCAAGGCATAC 3’ |
| GAPDH | F:5' CAAGGCTGTGGGCAAGGTCATC 3'  R:5’ GTGTCGCTGTTGAAGTCAGAGGAG 3’ |
